# Real-Time Electrical Resistivity Measurement and Mapping Platform of the Soils with an Autonomous Robot for Precision Farming Applications

**DOI:** 10.3390/s20010251

**Published:** 2020-01-01

**Authors:** İlker Ünal, Önder Kabaş, Salih Sözer

**Affiliations:** Department of Machine, Technical Science Vocational School, Akdeniz University, 07070 Antalya, Turkey; okabas@akdeniz.edu.tr (Ö.K.); sozer@akdeniz.edu.tr (S.S.)

**Keywords:** soil, soil electrical resistivity, autonomous robot, real-time measurement, precision farming, mapping

## Abstract

Soil electrical resistivity (ER) is an important indicator to indirectly determine soil physical and chemical properties such as moisture, salinity, porosity, organic matter level, bulk density, and soil texture. In this study, real-time ER measurement system has been developed with the help of an autonomous robot. The aim of this study is to provide rapid measurement of the ER in large areas using the Wenner four-probe measurement method for precision farming applications. The ER measurement platform consists of the Wenner probes, a *y*-axis shifter driven by a DC motor through a gear reducer, all installed on a steel-frame that mount to an autonomous robot. An embedded industrial computer and differential global positioning system (DGPS) were used to assist in real-time measuring, recording, mapping, and displaying the ER and the robot position during the field operation. The data acquisition software was codded in Microsoft Visual Basic.NET. Field experiments were carried out in a 1.2 ha farmland soil. ER and DGPS values were stored in Microsoft SQL Server 2005 database, an ordinary Kriging interpolation technique by ArcGIS was used and the average ER values were mapped for the soil depth between 0 and 50 cm. As a result, ER values were observed to be between 30.757 and 70.732 ohm-m. In conclusion, the experimental results showed that the designed system works quite well in the field and the ER measurement platform is a practical tool for providing real-time soil ER measurements.

## 1. Introduction

Soil electrical resistivity is an important indicator to indirectly determine the soil properties in the plant production because the suitable soil conditions and water are vital sources for plant root growth and solute transport, including plant nutrients and fertilizers [1]. The knowledge of soil resistivity is a valuable data in determining the composition of soil; such as for example, moisture [2], salinity [3], porosity [4], organic matter level [5], bulk density [6], and soil texture [7].

Soil investigation studies are commonly performed to determine the properties of the soil that involves topsoil and subsoil exploration such as physical mapping, soil sampling, and laboratory testing. Soil sampling and laboratory tests are usually performed to make subsoil investigation. Especially, the borehole method has been widely used to determine the soil properties due to its good data accuracy derived from the direct test method. However, this method has several difficulties and limitations such as high cost, time consuming, and insufficient data for huge farmlands. In this context, geophysical methods offer the chance to overcome some of the problems inherent in more conventional soil investigation techniques for soil structure characterization at larger spatial and temporal scales [8]. Soil is strongly correlated and can be quantified through the geoelectrical properties [9]. Moreover, the soil resistivity is an important property that is a geoelectrical quantity that measures how the soil reduces the electric current flow through it.

The soil composition is one of the most important factors affecting soil properties and has a heterogeneous structure consisting of solid, liquid, and gas phases. The solid and liquid phases are a determinative factor in soil electrical resistivity and behavior of electrical fields [10]. ER measurement was made of at the end of the 19th century by dipping two probes into the soil and measuring the voltage drop between two probes, which impinge a defined current into the soil. In this method, measurement results were incorrect as it intrinsically includes the sum of both soil resistivity and the contact resistivity between the probe and soil [11]. Wenner [12] suggested that the four-probe ER measurement method for minimizing contributions is caused by the soil-probe contact problems. ER measurement has been conducted with the four-probe method in soil studies since 1931 for evaluating soil moisture [13,14] and salinity [15,16] under field conditions. All the soil ER measurements applied in soil science are still based on the standard four-probe method since that time.

**Figure 1:**
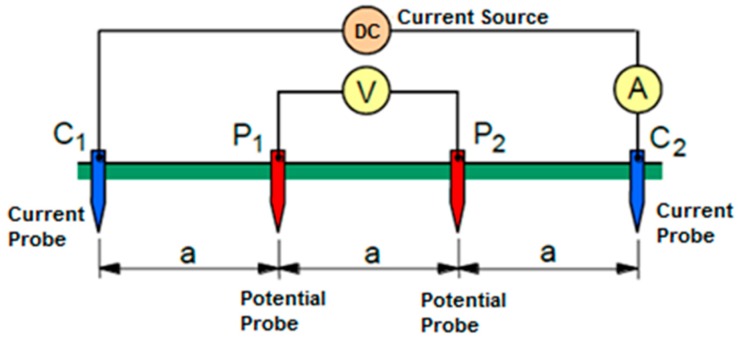
Four-probe Wenner configuration.

In the Wenner four-probe method, the system consists of four probes which are equally spaced (a) from each other to measure apparent soil ER [17]. The outer probes (C1 and C2) are used as the current source (I) for current injection to the soil and the inner probes (P1 and P2) are used as the voltage source to measure voltage difference (V) between the inner probes [12]. Wenner resistance (RW) between inner probes is calculated by dividing voltage by current. A Wenner configuration is shown in Figure 1. The apparent soil ER (*ρ_E_*) with this configuration is calculated by Equation (1):

(1) $$\;\rho_{E}\;=\;2*\pi *a*R_{W}$$

where: *ρ_E_* = measured apparent soil resistivity (ohm-m), *a* = probe spacing (m), *R_W_* = Wenner resistance (ohm). Four probes are dipped into the soil to be surveyed to a depth of not more than 1/20 the distance between the probes. The measured apparent soil ER value is average resistivity of the soil at a depth equivalent to the distance “*a*” between two probes. The measuring depth can be changed by changing the distance between the probes.

Today, agricultural production is carried out in huge farmlands and ER data should be collected fully automated to make a precision assessment of the farmland. Moreover, real-time soil resistivity measurement is important to map spatial farmland heterogeneity for precision farming applications. A useful approach for soil-property investigations is to use proximal soil measurements that combine soil sensors and data analysis methods to obtain high resolution soil data of the huge farmland [18]. Two portable systems are used to measure real time soil resistivity or conductivity of soil in agricultural studies—electrode-soil contact based system and noncontact electromagnetic induction (EM) system. The Veris (Veris Technologies, Inc., Salina, KS, USA) that has six coulter probes arranged in a Wenner method is the most important electrode–soil contact system to use to obtain multiple ER measurements representing different depths in agriculture [19]. The ARP (automatic resistivity profiling, Geocarta, France) is another Wenner based electrode–soil contact system using to acquire and process in real-time both electrical resistivity data and GPS information [20]. The other is the EM38 (Geonics Ltd., Mississauga, ON, Canada) that is the most widely used noncontact EM system in agriculture [21]. The electrode–soil contact-based system has the advantage that it does not require user setup configuration and measures different soil depth [22]. On the other hand, the non-contact EM system is lighter in weight, smaller in size, and thus easier to handle [23].

Real-time soil ER measurement is an important criterion for precision soil survey and can provide continuous measurements to determine temporal variables and soil structure over a huge farmland. The purpose of this study is the development and application of a real-time soil ER measurement system based on the four-probe Wenner method by the help of an autonomous robot for precision farming applications. Finally, for a thorough assessment of a measurement process, field study results are presented to identify the components of variation in the real-time soil ER measurement process.

## 2. Materials and Methods

The main objective of the designed system is to measure the apparent ER of the soil and map it. The system consists of four main parts:

1. Four-probes Wenner-based measurement platform: It is attached to an autonomous robot and it moves vertically to dip the Wenner probes in the soil. The Wenner probes are attached to the movable platform to measure the apparent ER values of the soil instantaneously.
2. Autonomous robot and steering algorithms: The autonomous robot is a four-wheel robot which steers with four DC motors. The differential steering mechanism is used. It can be steered point-to-point both autonomously and manually [24].
3. Data acquisition system: The system is used to collect data from a digital multimeter and a DGPS receiver on the measurement platform for the storing and mapping process.
4. Software development: The software is used to store data received from electronic instruments and to produce suitable database files to the mapping program.

### 2.1. Four-Probes Wenner-Based Measurement Platform

**Figure 2:**
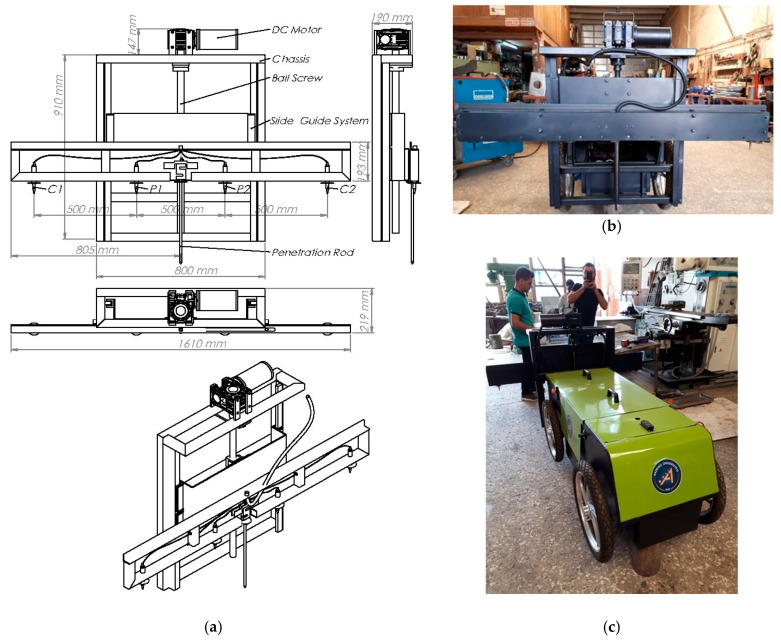
The developed measurement system. (**a**) Full scale technical drawing of the measurement platform; (**b**) full developed measurement platform; (**c**) four-wheel drive agricultural robot, which has the Wenner measurement system.

The developed measurement system is shown in Figure 2. The measurement platform was made of stainless steel. Some parts of the platform were made of the square steel tube 30 × 30 × 3 mm. The mechanical structure of the measuring system consists of two parts, called the H-shaped carrier grid and the Wenner measurement platform. For vertical movement of the measurement system, an H-shaped carrier grid was constructed by using two 30 × 30, 910 mm, and by three 30 × 30, 800 mm square steel tubes. Then, this grid was attached to the autonomous robot. The H-shaped grid held the Wenner measurement system. The H-shaped grid has two steel linear guides adjusted by 30 mm linear rail shaft guide supports and pillow blocks. The length of the linear guides is 910 mm. It was used a linear actuator, made up of a 30 × 850 mm ball screw, operated by a 24 V—500 W–1440 rpm DC motor which was coupled to a 1:40 reduction gearbox for the vertical movement. The DC motor was mounted on the H-shaped carrier grid. A 740 × 400 mm rectangular U-shaped sliding platform was attached to the H-shaped carrier grid. A square flanged nut ball screw is mounted on the back of the sliding platform. The ball screw was coupled to this square flange nut. Then, the Wenner measurement platform was connected to this rectangular U-shaped sliding platform.

The Wenner measurement platform has four Wenner probes. Four steel Wenner probes were linearly mounted on the Wenner measurement platform at 500 mm intervals to measure the average apparent ER values of the soil between 0–500 mm. The fiber isolation rings were used to ensure electrical isolation between the platform and the probes. The Wenner probes are 12 mm in diameter and 25 mm long. The probe length must be 1/20 of the distance between the probes. The distance between probes can be changed to measure apparent ER resistivity of the soil at the different depths. Each probe was wired with the insulated single core cable. The cables of the C1 and C2 probes are connected directly to a 24-volt battery to inject electric current into the soil. The cables of the P1 and P2 probes are connected to a multimeter to measure the potential difference between the probes in the soil. The system is also capable of measuring soil penetration resistance.

### 2.2. Autonomous Robot and Steering Algorithms

The four-wheel drive agricultural robot which is used in this study can be steered both autonomously and manually. Four 2.50 × 17 rubber wheels were chosen to steer the robot in field conditions. It has a differential steering mechanism. In this system, the speed difference between the right and left wheels of the robot can be created. To make the mobile robot steer in a straight line, speeds of the all wheels must be the same. If the speeds of the right and left wheels are different, the robot rotates to the slow wheels side. When the right and left wheels are rotated opposite each other, the mobile robot can be able to rotate 360 degrees where it is. The robot is powered by four 24 V—0.25 kW—1440 rpm DC motors which were coupled to a 1:10 reduction gearbox. Each wheel of the robot was independently coupled to motor-gearbox assemblies mounted on the robot chassis. In this way, the torque generated by the motors can be transmitted completely to the wheels. The robot’s weight is approximately 150 kg with batteries and the measurement system and the maximum speed is 20 km/h. Two RoboteQ FDC3260 three-channel DC motor control units (Roboteq Inc., Scottsdale, AZ, USA) were used to steer the robot by varying the speed and direction of the motors. The two 12 V-90 Ah rechargeable maintenance-free sealed batteries were used as the power source of the robot and other equipment’s. Moreover, two batteries were connected in the series to provide 24 V for the DC motors.

**Figure 3:**
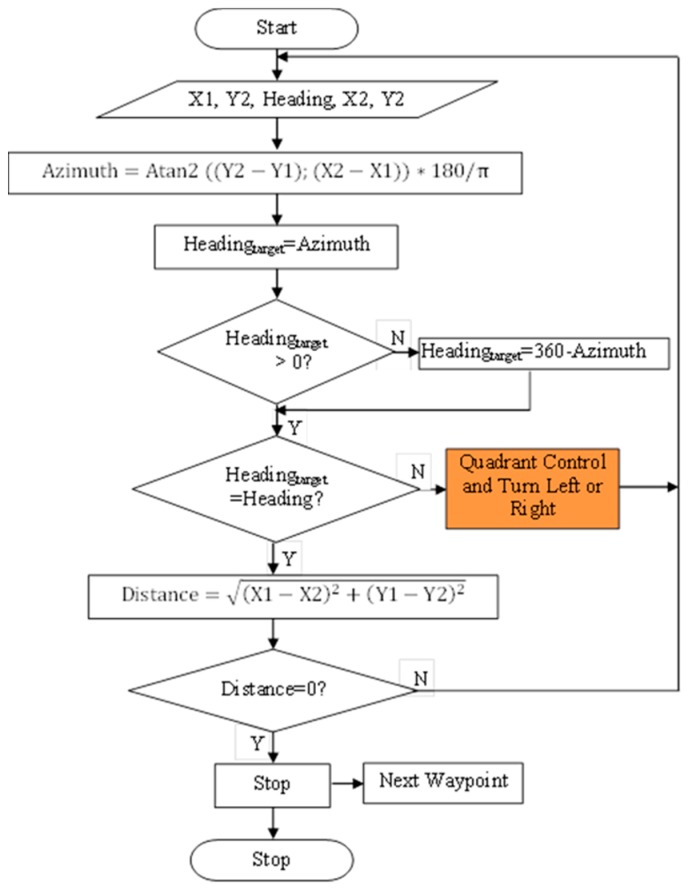
The flowchart for autonomous drive of the mobile robot.

**Figure 4:**
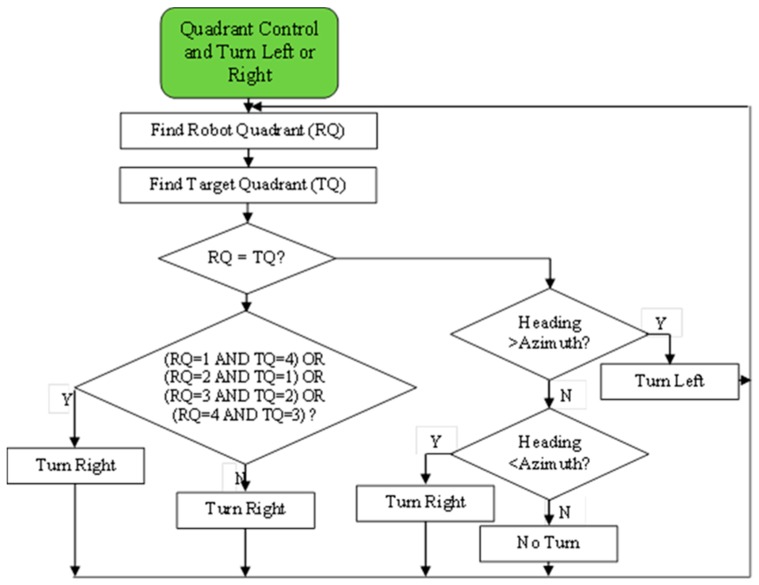
The flowchart of the quadrant control mechanism.

In order to operate the mobile robot both manually and autonomously, the navigation program was codded in Visual Studio.NET 2015 using Visual Basic.NET language. This program was firstly coded for two-wheel drive robots by the author in 2015 [24], and rearranged to the four-wheel drive robots for this study. However, the navigation algorithm is the same. The flowchart for autonomous drive of the mobile robot is given in Figure 3. The flowchart of the quadrant control mechanism is given in Figure 4.

In autonomous guidance algorithm, the angle difference is calculated using heading and azimuth angles. In this way, the robot can be steered to the desired direction. After that, the distance between the robot location and the target location is calculated. Lastly, when the heading angle is equal to the azimuth angle and the distance is equal to zero, the robot arrives at the desired location. In quadrant control, the compass dial is used to determine the quadrant in which the current point was located. The calculated azimuth angle helped in determining the quadrant in which the target point was located [24].

### 2.3. Data Acquisition System

The data acquisition system is described for two processes. The first is the autonomous steer system of the robot and the second is the measurement system of the apparent ER of the soil. In this study, Lilliput PC-700 industrial all-in-one touchscreen computer (Zhangzhou Lilliput Electronic Technology Co., Fujian, China) was used to manage and communicate with each other all of the electronic-based equipment placed on the robot. In the autonomous steer system, a Promark 500 RTK-GPS (Magellan Co., Santa Clara, CA, USA) receiver was used to collect geographical data. These data were used to determine the geographic location (latitude, longitude, speed, time, etc.) of the robot. In addition, the RTK-GPS receiver was used to determine the measurement location of the apparent ER of the soil for mapping. The Honeywell HMR3200 (Honeywell International Inc., Charlotte, NC, USA), which is a digital compass, was used to collect the precision heading angle of the robot for navigation software. The Protek 506 handheld digital multimeter (MCS Test Equipment Ltd., Denbighshire, UK) was used to measure voltage between P1 and P2 Wenner probes. Two RoboteQ FDC3260 three-channel DC motor control unit were used to control robot steering and also movement of the Wenner measurement platform. The RS232 protocol was used for connecting the industrial computer and other electronic devices.

### 2.4. Software Development

**Figure 5:**
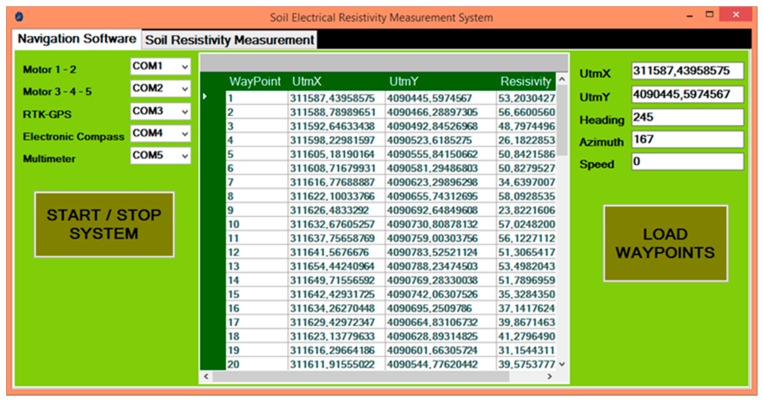
Navigation software.

**Figure 6:**
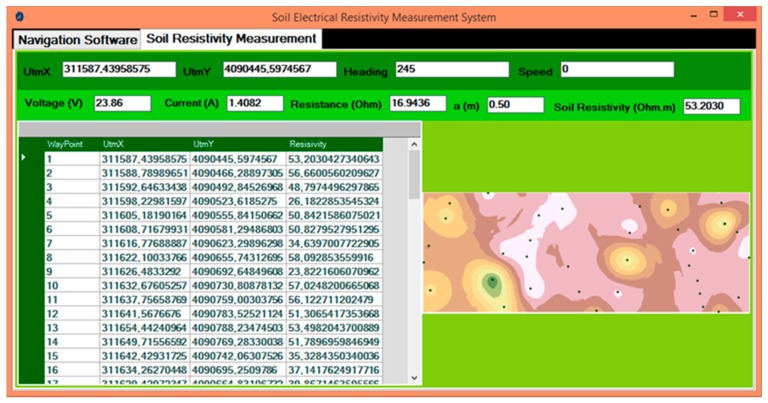
Soil resistivity measurement.

A program was developed using Microsoft Visual Basic.NET 2015 programming language to steer the robot both autonomously and manually and measure apparent ER values of the soil. It was used to control the robot and measuring system, monitor the telemetry data, store all the data in the database, and prepare the suitable file for ArcGIS mapping software. The program consists of two parts: Navigation software (Figure 5) and soil resistivity measurement (Figure 6).

In the navigation software, the waypoint file can be loaded from database into the program. In this way, the waypoints can be used to steer the robot from point to point for autonomous guidance. Each waypoint includes a longitude (X2) and latitude (Y2) value which is the location of the target point to be measured. There are two important angles for robot navigation: Robot’s heading angle and azimuth angle of the target point. Robot’s heading angle is taken from the HMR3200 electronic compass by the navigation software. Additionally, the azimuth angle is continuously calculated by the navigation software. Moreover, the distance between robot position (X1, Y1) and target position (X2, Y2) is calculated by the software. These calculations were shown in Figure 3.

In the soil resistivity measurement part, the current applied to the C1 and C2 probes and the voltage collected from probes P1 and P2 is monitored. Additionally, the Wenner resistance (Rw) is calculated instantly during the measurement using the obtained current and the measured voltage values. All data are stored instantly to the SQL Server 2005 database. The ArcObjects SDK 10 Microsoft .NET Framework was used to prepare and display the soil ER maps using ArcMap interface in ArcGIS.

### 2.5. Experimental Field and Data Collection

**Figure 7:**
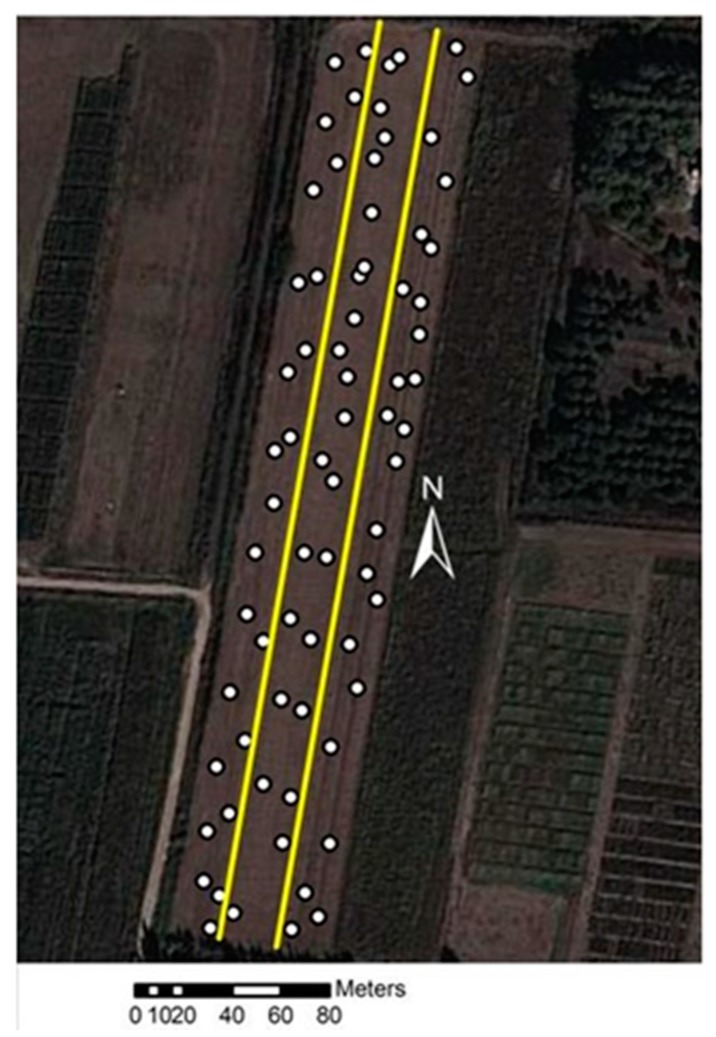
The experimental field.

The field experiments were carried out at the Batı Akdeniz Agricultural Research Institute, in Aksu, Antalya, Turkey (36°56′34.46″ N and 30°53′04.10″ E). The experimental field has an area of 1.2 ha and an elevation of 35 m above the sea level. In this field, the corn silage was harvested on July 25, 2019. The soil type is silty-clay, having a dark brown color, consists of 18% sand, 40% silt, and 42% clay. The organic matter content was 1.4%. Soil bulk density was 1.29 g/cm^3^, the water content was 6.8%, and the average soil penetration resistance value was 1.62 MPa between 0 and 20 cm. The experimental field was shown in Figure 7.

During the study within the experimental field, the robot was autonomously steered to 72 different geographical points and the average apparent soil ER values were collected for 0–50 cm depth. In this system, autonomous stop-and-go measurement method was used to measure the apparent soil ER values in large farmlands. The stop-and-go method is all about stopping the robot when taking the measurement. In this method, the agricultural robot goes to the first measurement point and stops, takes the measurement, and goes to the next measurement point. In this procedure, the digital map with high-resolution content of the experimental field was transferred into the ArcGIS 10.5 mapping software to determine the measurement points in large farmlands. In this way, a total 72 different GPS waypoints were randomly determined for autonomously steering the robot to the measurement point. All waypoints were stored to the database. After that, the agricultural robot was steered point-to-point to measure apparent soil ER value. All measured data were stored into the SQL Server 2005 database by the soil resistivity measurement software.

## 3. Results

In this study, the data obtained from all measured points were imported into Microsoft SQL Server 2005 database and mapped using ArcGIS 10.5 mapping software. In ArcGIS, ordinary Kriging interpolation was used to generate the contour map which makes a prediction of the apparent soil ER values in other parts of the experimental field for sampling.

The selection of the type of Kriging interpolation to use depends on the characteristics of the spatial data. Soil properties can spatially differ from point to point. As with most soil physical properties, soil is not also homogeneous in terms of electrical resistivity. In this regard, ordinary Kriging interpolation was used in this study. Simple Kriging is based on the theory of stationarity. This means that the mean and variance remain constant and are known in all locations. On the other hand, ordinary Kriging is a spatial estimation method and a linear geostatistical method that assumes that the mean may vary in the study area and does not remain constant. Universal Kriging is used to estimate spatial means when the data have a strong trend. This means that the trend is scale dependent. The apparent soil ER data may display trends over small geographic areas but at the scale of the huge farmlands, there is no trend that can be modeled by simple functions. Simple and universal Kriging interpolations were not chosen for this study because of these reasons.

**Figure 8:**
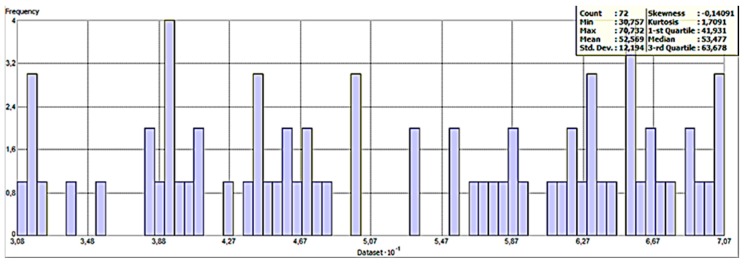
The histogram of the apparent soil ER (Electrical Resistivity) values.

A summary of the method used for Kriging interpolation is given in Table 1. The histogram of the apparent soil ER values is given in Figure 8. As can be seen in Figure 8, the minimum value of the soil ER is 30.757 ohm-m and the maximum value is 70.732 ohm-m. The skewness and Kurtosis values were observed as −0.14091 and 1.7091, respectively. Due to the skewness value being between −1 and −0.5, the data are reasonably skewed. This would mean that the sample data for the apparent soil ER are approximately symmetric. The Kurtosis value is low (<3). This means that the data are slightly platykurtic, the lack of outliers are in data, and the extreme values are less than that of the normal distribution.

**Figure 9:**
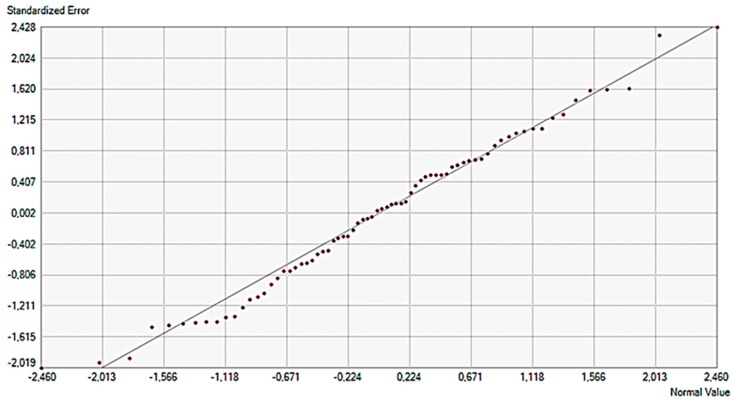
The normal QQ (Quantile – Quantile) plot graph of the standardized error.

The normal QQ plot graph was used to show the quantiles of the difference between the predicted and measured values and the corresponding quantiles from a standard normal distribution. As can be seen in Figure 9, the errors appear to be normally distributed even though there is a slight, possibly curved trend in the plot. Prediction errors of the ordinary Kriging method are given in Table 2.

**Figure 10:**
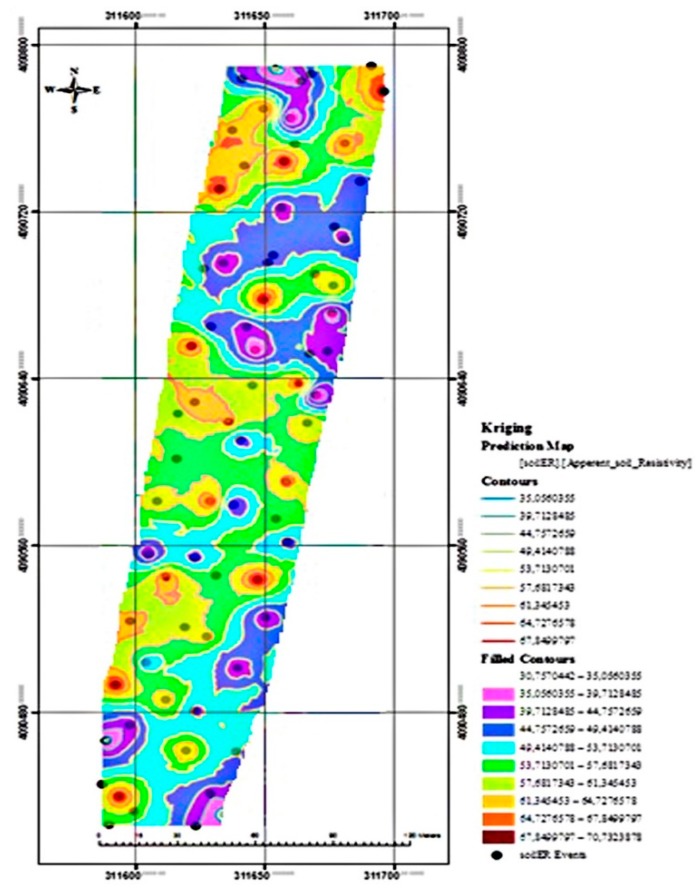
The interpolation map of the soil ER.

**Figure 11:**
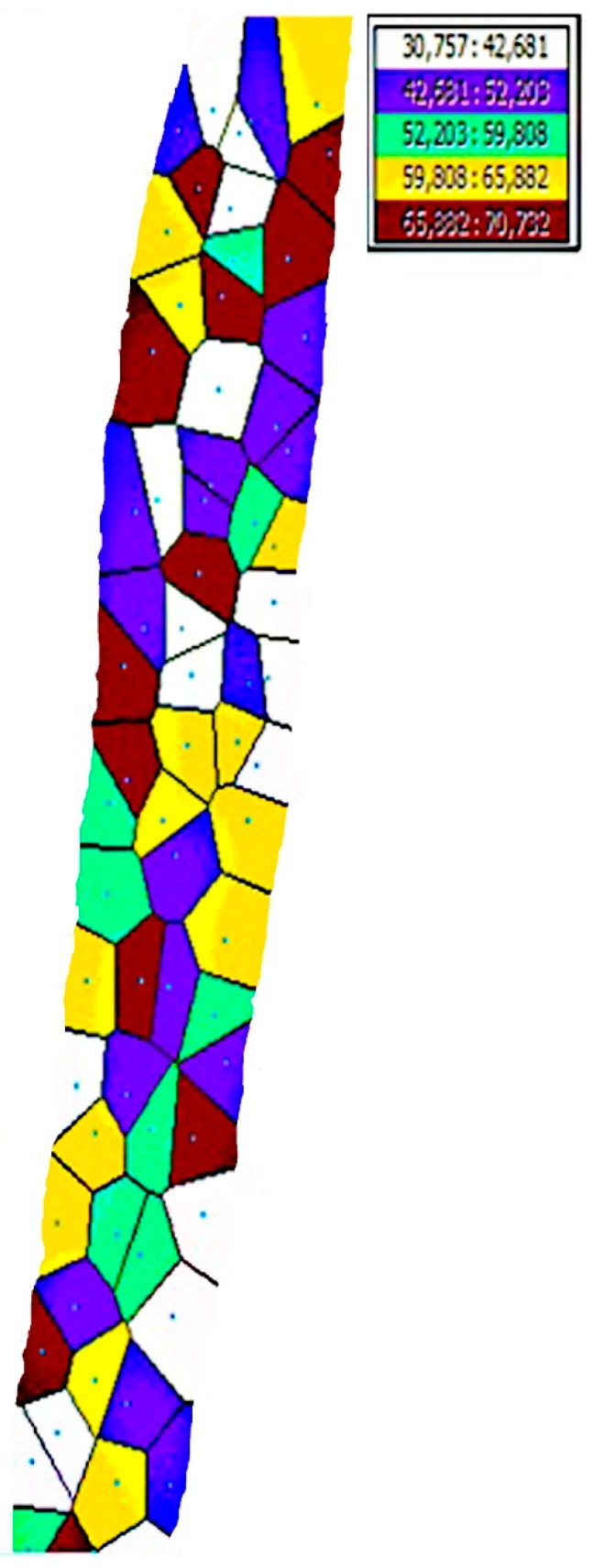
Voronoi map of the study.

In order to obtain the interpolation of the soil apparent ER values, the map of soil apparent ER on the experimental field was interpolated by using the ordinary Kriging approach. The interpolation map is given in Figure 10. Moreover, the Voronoi map of the study is given in Figure 11. It is observed that the ER values on the left side of the map are higher than the right side when the map is examined visually. This is also clearly seen on the Voronoi map of the study. If the measured points are close to each other, apparent soil ER values are approximately homogeneous. However, when the distance between the points increases, homogeneity decreases.

## 4. Discussion

The apparent soil ER values depend on several parameters such as size of the soil, porosity, and water content. Hunt [25] indicated that the electrical resistivity varies from 1.5 ohm-m and below for wet clay soils to more than 2400 ohm-m for massive and hard bedrocks (Table 3).

In the literature, there are not many studies measuring the apparent ER of the soil by the using mobile Wenner platform. However, there have been studies autonomously determining soil electrical resistivity of different soil types about soil science. Giao et al. [26] measured the electric resistivity of over 50 clay soil samples collected worldwide in the laboratory. Researchers have also measured the electric resistivity of over 50 soil samples taken from different locations in South Korea. As a result, they said that the sandy soil has a resistivity of above 10 ohm-m, the silty soil has a resistivity from 5 to 10 ohm-m. Juandia and Syahril [27] measured soil resistivity on 25 points across the study area using the Schlumberger configuration. Soil type was silty-sand. They reported that the average soil resistivity varied from 33 to 40.5 ohm-m. Rossi et al. [28] investigated the potential use of a direct current (DC) continuous resistivity profiling on-the-go sensor in precision viticulture. The authors used an automatic on-the-go DC recording resistivity meter (ARP, automatic resistivity profiling. Geocarta, Paris, France) in the three soil layers (V1 = 0–0.5, V2 = 0–1, and V3 = 0–2 m depth) on a vineyard area. Soil type was Inceptisol. The authors reported that soil ER values varied from 3–151 ohm-m for 0–0.5 m depth, 30–511 ohm-m for 0–1 m, and 9–750 ohm-m for 0–2 m depth. Lee and Yoon [29] investigated the theoretical relationship between elastic wave velocity and electrical resistivity. The authors measured the elastic wave velocity and electrical resistivity in several types of soils including sand, silty sand, silty clay, silt, and clay–sand mixture and the temperature compensated electrical resistivity probe was used for measuring. The authors said that the electrical resistivity showed at ranges of 1.23–2.17, 1.08–1.91, 1.01–1.40, 0.33–0.44, and 6.39–7.14 ohm-m in the order of soil types previously mentioned. Merritt et al. [30] developed a methodology for measurement and modeling of the moisture–electrical resistivity relationship of fine-grained unsaturated clay-based soils and electrical anisotropy. Soil resistivity measurements were conducted for four different soil types: Silty-clay, fine sand, clayey sandy silt, and siltstone. The results showed at approximately ranges of 10–100, 100–150, 50–800, 100–10000 ohm-m. The authors reported that the soil resistivity increases with decreasing moisture content. Kim et al. [31] evaluated the effects of soil properties and electrical conductivity on the water content reflectometer calibration for landfill cover soils. For this aim, the electrical conductivity measurements were performed for a set of 28 soils which have different soil textures by using high-frequency time domain reflectometer (TDR). The authors reported that the soils with a greater clay content or organic content have higher electrical conductivity than the soils with silts and sands. This means that the ER values of the clay soils should be low.

The traditional soil sampling is made by using the borehole method to determine the soil physical, chemical, or biological properties of soil layer in laboratory conditions. In this method, the laboratory calibration of the soil ER with soil moisture should be done. However, laboratory calibration may not give the correct relationship between soil moisture and electrical resistivity for real soil conditions [32]. On the other hand, in the precision farming domain, the autonomous and continuously measurement of the apparent soil ER has some advantages such as fast measurement and low cost for mapping both the horizontal and the vertical spatial variability in large farmlands. Moreover, the user setup configuration or calibration in this system is not required. Both Veris and ARP systems have been developed to achieve these advantages and measure the soil resistivity or conductivity as mobile for precision farming applications. The cost of the basic Veris system is 11.500 USD [33]. However, there is not any price about the ARP system in the literature. However, Andrenelli et al. [34] reported that the daily cost is 3000 Euro for ARP system usage. In our proposed system, the study was carried out within a project and the total budget was 8000 USD for all the system.

Both the ARP and the Veris system are semi-mounted type measuring platforms that are attached to the tractor or any other vehicle such as ATV. In this context, these systems need a traction system and at least one operator for their operation. Moreover, these systems are not lightweight and have low maneuverability. On the other hand, the benefits of the designed system are obvious: Easy to manufacture, compact measurement system with robot, lightweight, low manufacturing and operation cost, high maneuverability, and used autonomously.

This study was undertaken on silty-clay type soil by using the measurement system developed by us. In this study, the apparent soil ER values were measured between 30.757 and 70.732 ohm-m. The measurement results were shown similarity with the abovementioned literatures for silty-clay soils [25,27,30]. In addition to our results, for the obtained apparent soil ER values to be more significant, soil penetration resistance should be simultaneously measured and correlated with soil moisture content and bulk density [35,36,37,38,39,40,41,42]. No faults were detected in the electromechanical, data acquisition, and software parts of the system during field operation. The experimental results showed that our measurement system is suitable for map-based precision farming applications.

## 5. Conclusions

In this study, a new design for real-time apparent soil ER measuring system and its mapping capability has been presented for map-based precision farming applications. Although the laboratory analysis is usually the reliable method for determining most soil properties, real-time measurements to monitor the soil properties have advantages and benefits for precision farming applications. The DC apparent soil ER measurement method is one of the simplest geophysical techniques and still employed extensively because of its easy-to-use, no calibration required, and relatively easy interpretation in all engineering studies. However, a robot-based mobile Wenner measurement platform has not been found in the agriculture literature. The apparent soil ER map created by the developed software can be a useful source for precision farming applications across different fields. For researchers, data collection, analysis, and interpretation from farmlands have always been hard, time-consuming, and tedious studies in agricultural applications. The results of the study show that using this system is important for researches and professional applications of soil science.

## Figures and Tables

**Table 1:** The field study data.

Method	
Name	Kriging
Type	Ordinary
Output Type	Prediction
Neighbors to Include	5
Include at Least	2
Sector Type	Four and 45 degree
Major Semiaxis	21.669411886777
Minor Semiaxis	21.669411886777
Angle	0
Variogram	Semivariogram
Number of Lags	12
Lag Size	2.429475299987
Model Type	Stable
Parameter	1.131640625
Range	21.669411886777
Anisotropy	No
Partial Sill	164.681994538028

**Table 2:** Prediction errors of the ordinary Kriging method.

Prediction Errors (ohm-m)	
Number of Samples	72
Mean	−0.2602
Root Mean Square	13.1404
Mean Standardized	0.01682
Root Mean Square Standardized	1.02197
Average Standard Error	12.7975
Regression Function (Predicted)	−0.0133 * x + 52.8556
Regression Function (Error)	−1.0133 * x + 52.8556
Regression Function (Standardized Error)	−0.0747 * x + 3.89899

**Table 3:** ER values of the different soil types.

Materials	Resistivity (ohm-m)
Clayey soils: Wet to moist	1.5–3.0
Silty clay and silty soils: Wet to moist	3–15
Silty and sandy soils: Moist to dry	15–150
Bedrock: Well fractured to slightly fractured with moist soil-filled cracks	150–300
Sand and gravel with silt	About 300
Sand and gravel with silt layers	300–2400
Bedrock: Slightly fractured with dry soil-filled cracks	300–2400
Sand and gravel deposits: Coarse and dry	>2400
Bedrock: Massive and hard	>2400
